# Increased FGF1-FGFRc expression in idiopathic pulmonary fibrosis

**DOI:** 10.1186/s12931-015-0242-2

**Published:** 2015-07-03

**Authors:** BreAnne MacKenzie, Martina Korfei, Ingrid Henneke, Zaneta Sibinska, Xia Tian, Stefanie Hezel, Salma Dilai, Roxana Wasnick, Beate Schneider, Jochen Wilhelm, Elie El Agha, Walter Klepetko, Werner Seeger, Ralph Schermuly, Andreas Günther, Saverio Bellusci

**Affiliations:** German Center for Lung Research, Excellence Cluster Cardio-Pulmonary System, Universities of Giessen and Marburg Lung Center, Giessen, Hessen Germany; Department of Thoracic Surgery, General Hospital University Vienna, Vienna, Austria; German Center for Lung Research, Greifenstein, Germany; AGAPLESION Lung Clinic Waldhof-Elgershausen, Greifenstein, Germany; Developmental Biology Program, Division of Surgery, Saban Research Institute of Children’s Hospital Los Angeles, University of Southern California Keck School of Medicine, Los Angeles, CA USA; Institute of Fundamental Medicine and Biology, Kazan (Volga Region) Federal University, 18 Kremlyovskaya Street, Kazan, 420008 Russian Federation

## Abstract

**Background:**

Recent clinical studies show that tyrosine kinase inhibitors slow the rate of lung function decline and decrease the number of acute exacerbations in patients with Idiopathic Pulmonary Fibrosis (IPF). However, in the murine bleomycin model of fibrosis, not all tyrosine kinase signaling is detrimental. Exogenous ligands Fibroblast Growth Factor (FGF) 7 and 10 improve murine lung repair and increase survival after injury via tyrosine kinase FGF receptor 2b-signaling. Therefore, the level and location of FGF/FGFR expression as well as the exogenous effect of the most highly expressed FGFR2b ligand, FGF1, was analyzed on human lung fibroblasts.

**Methods:**

FGF ligand and receptor expression was evaluated in donor and IPF whole lung homogenates using western blotting and qPCR. Immunohistochemistry for FGF1 and FGFR1/2/3/4 were performed on human lung tissue. Lastly, the effects of FGF1, a potent, multi-FGFR ligand, were studied on primary cultures of IPF and non-IPF donor fibroblasts. Western blots for pro-fibrotic markers, proliferation, FACS for apoptosis, transwell assays and MetaMorph analyses on cell cultures were performed.

**Results:**

Whole lung homogenate analyses revealed decreased FGFR b-isoform expression, and an increase in FGFR c-isoform expression. Of the FGFR2b-ligands, FGF1 was the most significantly increased in IPF patients; downstream targets of FGF-signaling, p-ERK1/2 and p-AKT were also increased. Immunohistochemistry revealed FGF1 co-localization within basal cell sheets, myofibroblast foci, and Surfactant protein-C positive alveolar epithelial type-II cells as well as co-localization with FGFR1, FGFR2, FGFR3, FGFR4 and myofibroblasts expressing the migratory marker Fascin. Both alone and in the presence of heparin, FGF1 led to increased MAPK-signaling in primary lung fibroblasts. While smooth muscle actin was unchanged, heparin + FGF1 decreased collagen production in IPF fibroblasts. In addition, FGF1 + heparin increased apoptosis and cell migration. The FGFR inhibitor (PD173074) attenuated these effects.

**Conclusions:**

Strong expression of FGF1/FGFRs in pathogenic regions of IPF suggest that aberrant FGF1-FGFR signaling is increased in IPF patients and may contribute to the pathogenesis of lung fibrosis by supporting fibroblast migration and increased MAPK-signaling.

**Electronic supplementary material:**

The online version of this article (doi:10.1186/s12931-015-0242-2) contains supplementary material, which is available to authorized users.

## Introduction

Idiopathic pulmonary fibrosis (IPF) is a rare interstitial lung disease of unknown origin, with prevalence rates ranging from 2-4/10000 [1]. Diagnosis usually occurs in the sixth and seventh decades of life and incidence appears to be rising in older males [2]. Despite the recent approvals of Pirfenidone in Europe [3], and the tyrosine kinase inhibitor, Nintedanib [4] in both Europe and the USA, IPF is still characterized by 5-year survival rates that approximate 10-15 % [1].

Both Fgf7 and Fgf10 are mesenchymal-derived growth factors that signal in a paracrine manner to bind with high affinity to epithelial expressed Fgfr2 b-isoform [5]. Overexpression or administration of exogenous fibroblast growth factors (Fgf)-7/10 [6, 7] diminishes the extent of epithelial injury and apoptosis thereby attenuating bleomycin-induced lung fibrosis in rodents. In addition, attenuation of the mesenchymal expressed c-isoform of Fgfr2 receptor led to a reduction in bleomycin-induced fibrosis [8]. Thus, in the bleomycin model of lung fibrosis, enhanced Fgfr2b-isoform signaling may confer epithelial repair and survival, while c-isoform Fgf-signaling may support or maintain fibrosis. Fgf1, also known as heparin binding growth factor, or acidic Fgf, is expressed by both mesenchymal and epithelial cell types in the lung [9] and binds with high affinity to all Fgfrs [10]. Thus, Fgf1 may play multiple roles during lung repair as it signals to Fgfrs expressed by both epithelial and mesenchymal cell types.

Fgf/Fgfr-binding is stabilized by heparin and transduced by a phosphorylation cascade, mediated by fibroblast growth factor receptor substrate (Frs2) [11] which activates PI3k and Mapk-signaling pathways and/or activation of phospholipase C gamma (Plc-γ) [12]. Signaling culminates in survival, growth and/or differentiation of cells depending on the context. Fgf-Fgfr induced Mapk-signaling is negatively regulated by Spry2 [13–15] and Spry4 [16], both of which are activated by Erk and inhibit the Mapk pathway by binding to the Mapk kinase, Raf. Etv4, also known as Pea3, is also a target of Fgf-signaling [17]. Increased Etv4 expression is associated with increased cell invasion [18] and metastasis in lung cancer [19].

Until now, studies investigating the activity of heparin + FGF1 in the context of lung fibrosis have been carried out exclusively on cell lines. Treatment of lung fibroblast cell-line N12, with heparin + FGF1 had no impact on proliferation but induced apoptosis and decreased smooth muscle actin production [20]. Moreover, FGF1 in the presence of heparin reversed TGF-beta1-mediated epithelial to mesenchymal transition (EMT) of A549 and RLE-6TN epithelial cell lines [21]. Taken together, these studies implicate FGF1 as an anti-fibrotic factor. This is the first study to investigate the level and location of FGF1 and FGFRs using IPF and donor (non-IPF) lung tissues. This study revealed strong expression of FGF1 and FGFR receptors in pathogenic areas characteristic of IPF including basal cell sheets and fibroblastic foci. In addition, FGF1 and FGFRs co-localized with the previously described cell invasion marker, Fascin [22]. Therefore, despite evidence from previous publications, this study hypothesized that aberrant, increased FGF1-FGFR signaling contributes to lung remodeling in IPF. Contrary to studies performed previously on N12 fibroblast cell lines, no significant changes in smooth muscle actin production was detected in primary lung fibroblasts treated with heparin + FGF1. However, a trend towards decreased collagen 1a1 production was observed. In agreement with these studies [20], primary lung fibroblasts isolated from end-stage IPF patients and treated with FGF1 in the presence of heparin showed no change in proliferation but displayed increased apoptosis. Upon further analyses, transwell migration assays as well as MetaMorph analyses of cell motility indicated increased migration in fibroblasts exposed to FGF1 + heparin. These effects were attenuated in the presence of an FGFR-signaling inhibitor; PD173074. These results suggest that FGF1-FGFR-signaling in the presence of heparin may contribute to lung remodeling by enhancing invasive capabilities of fibroblasts. Moreover, these results illustrate the potentially dual nature of FGF1-signaling in the lung and may indicate a mechanism by which endogenous FGF1-signaling plays both a protective (epithelial cell survival and fibroblast apoptosis) and pathological role (fibroblast invasion) in IPF.

## Material and methods

### Ethics statement

The study protocol for tissue donation was approved by the Ethics Committee of the Justus-Liebig-University School of Medicine (AZ 111/08). Informed consent was obtained from each individual patient or next of kin.

### Human tissue

Lung homogenates from IPF (*n* = 36) or donors (*n* = 15) were obtained during transplantation at the Department of Cardiothoracic Surgery, University of Vienna, Austria or University Hospital Giessen-Marburg, Giessen, Germany and processed as previously described [3].

### Western blot

Loading buffer was added to protein samples from cell extracts (5 % SDS in bromophenol blue and β-mercaptoethanol) denatured for 5 min at 95 °C and cooled on ice. At least 10 μg of sample was loaded on a 10 % polyacrylamide gel and run at 25 mA for 2 h then transferred to a polyvinylidene fluoride (PVDF) membrane (Amersham) by semi-dry electro-blotting (70 mA per gel; gel size: 7×9 cm) for 90 min. The membrane was blocked with 5 % milk in TBS-blocking buffer at RT for 1 h followed by 4 °C incubation with primary antibody overnight. Membranes were washed in 1X TBST buffer four times and incubated with HRP-labeled secondary antibody at RT for 1 h followed by washing with 1X TBS-T. Bands were detected by ECL (Enhanced Chemi-luminescence, Amersham, Germany) treatment, followed by exposure of the membrane. (Antibody information is available in Additional file 1: Supplementary Methods).

### Quantitative PCR

RNA was reverse-transcribed (Qiagen QuantiTect Reverse Transcription Kit (205313). cDNA was diluted to 20 ng/μL. Primers were designed to span introns using Roche Applied Sciences online Assay Design Tool. Sybr Green Master Mix (Applied Biosciences 4309155) was used for qPCR with a Roche LightCycler480 machine. Samples were run in triplicates using *PGBD* as a reference. Primers are available in Additional file 1: Supplementary methods.

### Immunohistochemistry (IHC)

Human lungs were placed in 4 % (w/v) paraformaldehyde for 12-24 h, and processed for paraffin embedding. 3 μm sections were cut and mounted on slides (Super Frost Plus, Langenbrinck). Paraffin-embedded tissue sections of donor and IPF-lungs were deparaffinized in xylene and rehydrated in graded alcohol. Antigens were retrieved by microwave antigen retrieval (800 W); in 10 mmol/L freshly prepared citrate buffer (pH 6.0) for 5 min. For immunostaining, the streptavidin-biotin-alkaline phosphatase (AP) - or the streptavidin-biotin- horseradish peroxidase (HRP) method was used with the ZytoChem-Plus AP Kit (Fast Red), Broad Spectrum (Zytomed Systems, Berlin, Germany) according to the manufacturer’s protocol. Sections were counterstained with hemalaun (Mayers hemalaun solution, WALDECK Division CHROMA GmbH & CO KG, Münster, Germany) and mounted in Glycergel (DakoCytomation). Control sections were treated with 2 % BSA in PBS alone or with rabbit or mouse primary antibody isotype control (#_NB810-56910 and #_AM03096PU-N, Acris Antibodies GmbH, Germany) to determine the specificity of the staining. Lung tissue sections were scanned with a Mirax Desk slide-scanning device (Mirax Desk, Zeiss, Germany), and examined histo-pathologically at 50×, 100×, 200×, 400×. IHC for mentioned antibodies was performed in at least 8 IPF and 5 control-lung samples. A complete list of antibodies and dilutions is provided in the online supplement.

### IPF and non-IPF fibroblast cell culture

5 cm lung cubic biopsies of human lung tissue were washed in PBS and cut into small pieces in growing culture medium: Dulbecco's Modified Eagle's Medium (DMEM) with 10 % Fetal calf serum (FCS) 1 % glutamine, and 1 % Penicillin-Streptomycin. Pieces were seeded initially in a large 75 cm^2^ flask and grown out at 37 °C, 5 % CO_2_ for up to one month with weekly media changes according to a previously published protocol [23]. No enzymatic digestion was performed. Non-attached cells were washed away, and adherent fibroblasts remained. After the second passage, fibroblasts were frozen in 10 % DMSO, 10 % FCS and DMEM and stored in liquid nitrogen. Cells were thawed, seeded and treated with human recombinant FGF1 (R&D Systems #231-BC-025) with or without heparin (Sigma #H3149) or FGFR inhibitor PD173074 (Tocris Bioscience #3044).

### Annexin V FACS assay

The affymetrix APC annexin V and propidium iodide staining kit was used to perform an apoptosis assay according to manufacturers instructions (eBioscience #88-8007-74) on an Accuri C6 flow cytometer (BD Biosciences). Briefly, cells were washed twice in cold PBS, then trypsinized at 37 °C for 5 min. 2 wells of fibroblasts grown in 6-well plates were gently collected, combined and pelleted. Pellets were washed in PBS, followed by binding buffer, and incubated first with kit antibody, then propidium iodide in the dark for 10 min. At least 30,000 cells were counted per FACS experiment. Gating was established based on plots of propidium iodide alone and annexin V alone.

### Transwell assay

Primary lung fibroblasts were starved for 24 h and seeded (12,000 cells/well) in the upper chamber of the transwell (6.5-mm transwell inserts with 8.0 μm pore size polycarbonate membrane CLS3422-48EA, Sigma Aldrich) containing serum free DMEM F-12 medium and the lower chambers with various experimental conditions. The system was incubated at 37 °C, 5 % CO2 to allow the migration of cells through the membrane (8.0 μm). After 16 h, media was removed using a gentle suction and cells were washed with 1X PBS, fixed with methanol and stained with crystal violet. Next, the transwell was swabbed to remove the non-migrated cells and the total number of migrated cells was quantified using phase contrast microscope.

### MetaMorph analyses of cell motility

Fibroblasts from at least 3 biologically unique samples were seeded at low density, in a 24-well-plate, starved 24 h, and underwent various treatments. The plate was set on a motorized stage in a 5 % CO_2_ and 37 °C environment. Random regions were marked, and Leica Live Imaging Software snapped a photo of these regions every 5 min for 20 h. LIF files were exported and analyzed in Leica MetaMorph software version 1.5.0. The average total distance traveled of at least 6 random cells per group were tracked and recorded.

### Statistical analyses

A Student’s *t*-Test was performed on the log-transformed value of the qPCR fold changes. For western blots, t-tests were performed on the probit values. One-way ANOVAs with Dunnet’s test (untreated groups served as controls) were performed on Transwell and MetaMorph data.

## Results

### FGF1-FGFR1/2/3 as well as downstream targets PI3K- and MAPK-signaling were increased in whole lung homogenates of end-stage IPF patients

**Fig. 1:**
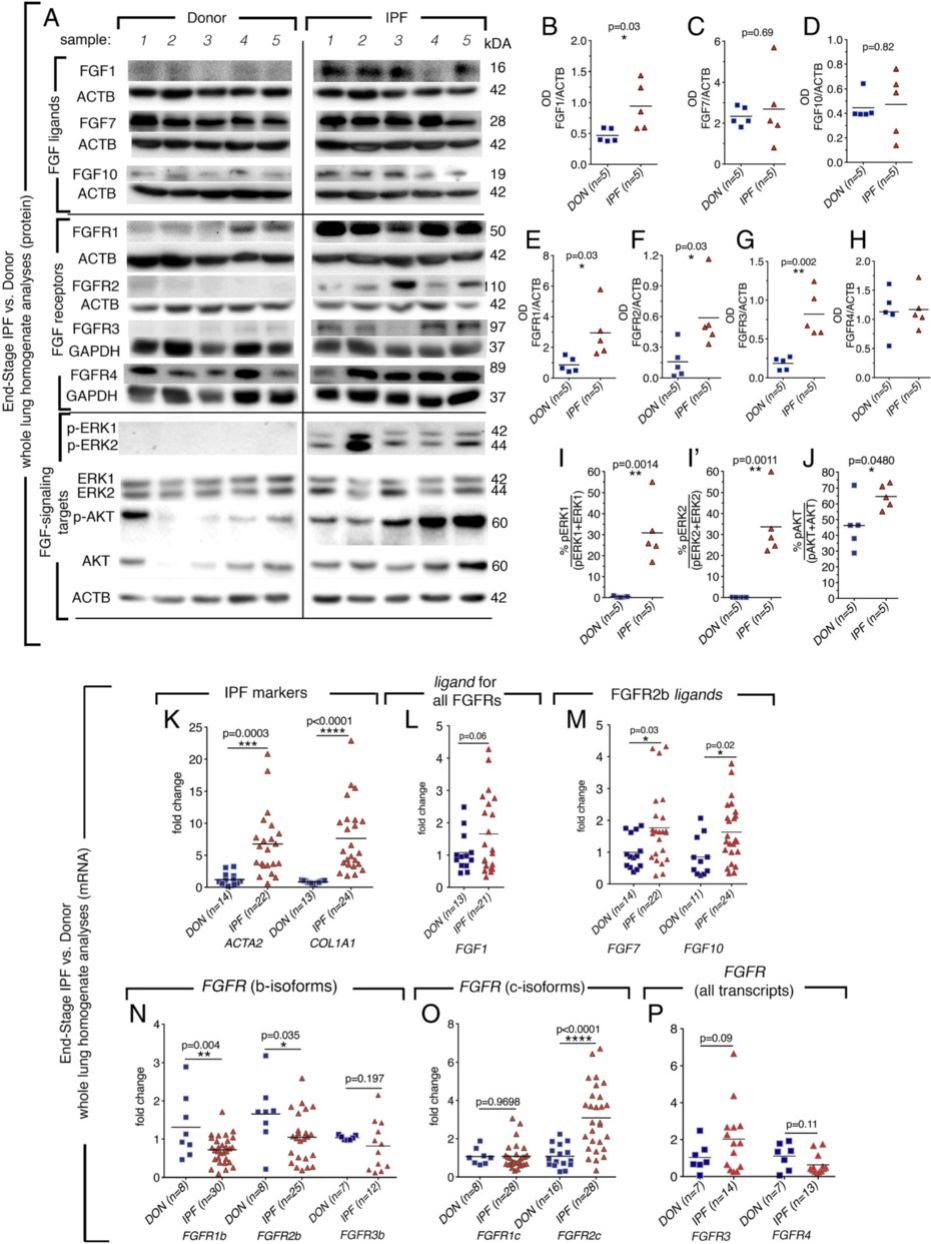
Western blot and qPCR analyses of IPF and donor whole lung homogenate lysates and RNA revealed increased FGF1-FGFR expression. Western blots were performed on end-stage IPF (IPF) and non-IPF, donor lung homogenate lysates for FGF, MAPK and PI3/K signaling markers (**a**). Densitometry plots of arbitrary units indicated a significant increase in FGF1 (**b**), no increase in FGF7 (**c**), FGF10 (**d**). Receptors FGFR1 (**e**), FGFR2 (**f**), FGFR3 (**g**) but not FGFR4 (**h**) were increased in IPF samples as well as p-ERK1 (**i**), p-ERK2 (**i**’) and p-AKT (**j**). *ACTA2* and *COL1A1* transcripts were increased in IPF samples (**k**) as well as FGFR2b ligand transcripts: *FGF1, FGF7,* (**l**) and *FGF10* (**m**). B-isoforms of FGFRs were decreased (**n**) while *FGFR2* c-isoform was significantly increased (**o**). Expression of FGFR3 was variable and FGFR4 (**p**) was not changed between IPF and donor

Western blots were performed for FGFR2b ligands FGF1*,* FGF7*,* and FGF10*.* A significant increase in FGF1 was observed in IPF patients (Fig. 1a,b). FGF7 and FGF10 protein levels were not significantly different (Fig. 1a,c,d). In addition, FGFR1 (Fig. 1a,e), FGFR2 (Fig. 1a,f) and FGFR3 (Fig. 1a,g) were upregulated in IPF lungs, while FGFR4 was unchanged. Next, downstream pathways activated by growth factor signaling, including FGFR-signaling, including: activated protein kinases (MAPK) and phosphatidylinositol-4,5-bisphosphate 3-kinase (PI3K), [24] were analyzed. Both p-ERK1 and p-ERK2 were increased in IPF samples (Fig. 1a, I-I’) as well as, p-AKT (Fig. 1a,j). In addition, qPCR was performed on FGF ligands and receptor transcripts. IPF samples showed characteristic increases in *Smooth muscle actin* (*ACTA2*) and *Collagen 1a1* (*COL1A1*) transcripts (Fig. 1k). A trend towards an increase in *FGF1* transcript in IPF lungs was observed (Fig. 1l). *FGF7* and *FGF10* transcripts were also increased (Fig. 1m). While the overall trends in the direction of the changes of expression were similar, mRNA transcription profiles did not always correlate exactly with the protein expression profiles. Post-transcriptional regulation of RNA by microRNAs and/or heterogeneous homogenates may account for the discrepancies. As antibodies used against FGFR receptors were not isoform specific, qPCR was performed to determine which isoforms of *FGFRs* were increased. Epithelial b-isoform expression of *FGFR1 and 2* were decreased in IPF homogenates while *FGFR3b* transcript expression was variable (Fig. 1n). The mesenchymally expressed c-isoform of *FGFR2* and to some extent *FGFR3*, were increased while the expression of *FGFR1c* and *FGFR4* were unchanged (Fig. 1o,p). These data suggest that despite the increase in *FGF7* and *FGF10*, which have been shown to attenuate lung injury in mice, the low level of FGFR2b receptor suggests that epithelial FGFR2b-signaling may be reduced in IPF patients. In contrast, the abundant expression of FGFR c-isoform, and availability of FGF1 ligand suggested that FGF1-FGFRc signaling may be increased in IPF patients.

### Immunohistochemistry on serial sections revealed co-localization of FGF1 with both epithelial and mesenchymal derived cells in pathogenic regions of IPF as well as co-localization with FGFR1, FGFR2, FGFR3, FGFR4, and Fascin

**Fig. 2:**
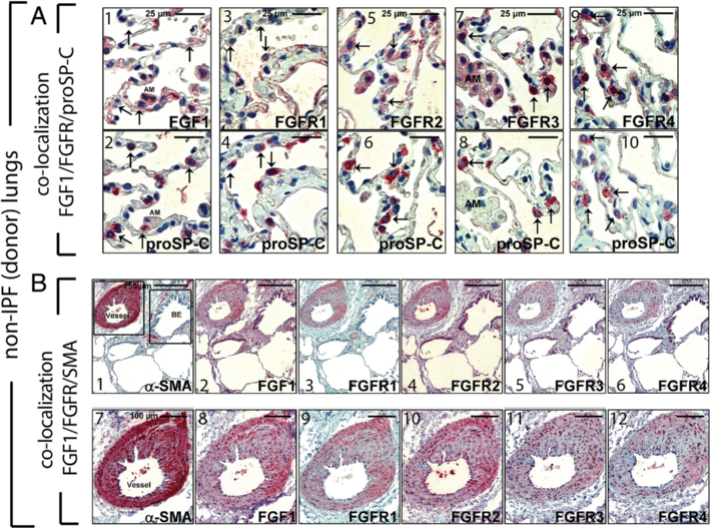
Expression and localization of FGF1 and FGF-receptors FGFR1, FGFR2, FGFR3 and FGFR4 in non-IPF, donor control lungs. Representative immunohistochemistry for FGF1 (**a**1), FGFR1, FGFR2, FGFR3, and pro-surfactant protein C (proSP-C) in serial sections of normal control-lung tissue. Alveolar epithelial type-II cells (AECII, indicated by arrows) of normal lungs do not express FGF1 (**a**1,2) and FGFR1 (**a**3,4), but indicate moderate expression of FGFR2 (**a**5,6) and robust expression of FGFR3 (**a**7,8), and FGFR4 (**a**9,10). Representative immunohistochemistry for FGF1, FGFR1, FGFR2, FGFR3, and alpha-smooth-muscle-actin (α-SMA) in serial sections of normal control-lung tissue. Vascular smooth muscle cells (upper and middle panels) showed strong expression of FGF1 (**b**2,8) and FGF-receptors FGFR1 (**b**3,9), FGFR2 (**b**4,10), FGFR3 (**b**5,11), and FGFR4 (**b**6,12). Scale bars: a1-10 (25 μm); b1-6 (250 μm), b7-12 (100 μm)

Non-IPF alveolar epithelium revealed faint, expression of FGF1 in pro-SP-C+ alveolar epithelial type II cells (AECII, Fig. 2; a1-2). FGFR1 staining was absent, and (Fig. 2, a3-4)FGFR2 indicated moderate expression in normal proSP-C+ AECII cells (Fig. 2; a5-6) whereas FGFR3 and FGFR4 were robustly expressed by this cell type (Fig. 2; a7-8 and a9-10, respectively). The co-localization of FGF1 and FGFR with spindle-shaped, α-SMA positive cells was observed (Fig. 2; b1-6). Alveolar macrophages of normal donor lungs also stained positive for FGF1 as well as for FGFR1-4 (Additional file 2: Figure S1).

FGF1 co-localized with α-SMA+ vascular smooth muscle cells (VSMCs) of donor lungs (Fig. 2; b2,8 vs. B1,7). In addition, FGFR1 (Fig. 2; b3,9 vs. B1,7) and FGFR2 stained VSMCs (Fig. 2; b4,10 vs. b1,7) as well as FGFR3 and FGFR4 (Fig. 2; b5,11 and b6,12, respectively).

**Fig. 3:**
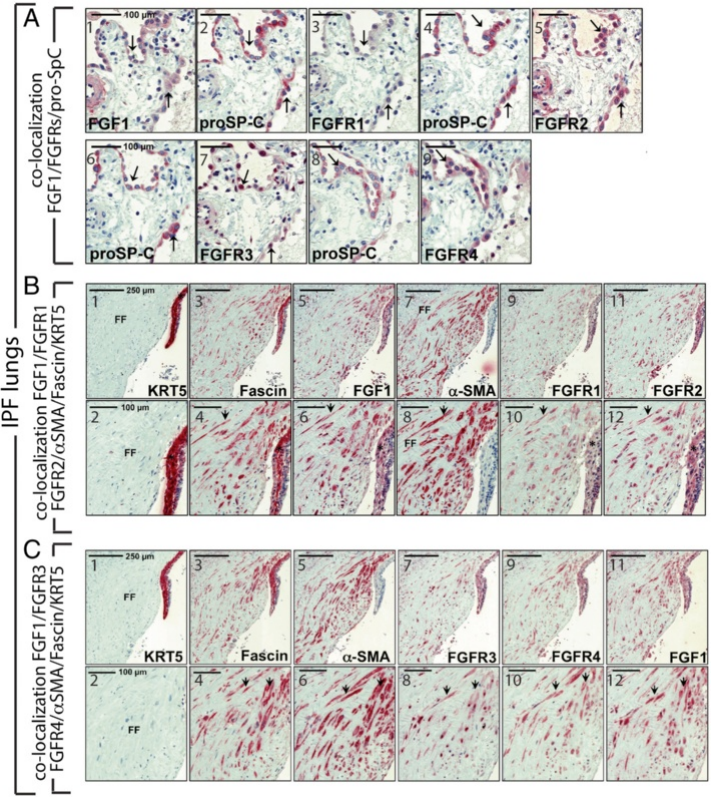
Expression and localization of FGF1 and FGF-receptors FGFR1, FGFR2, FGFR3 and FGFR4 in hyperplastic, overlying alveolar epithelium, fibroblastic foci and basal cell sheets in idiopathic pulmonary fibrosis (IPF) lungs. **a.** Representative immunohistochemistry for FGF1, FGFR1, FGFR2, FGFR3, and prosurfactant protein C (pro-SP-C) in serial sections of IPF lung tissue. Alveolar epithelial type-II cells (AECII, indicated by arrows) of IPF lungs express FGF1 (a1,2), FGFR2 (a4,5), FGFR3 (a6,7), and FGFR4 (a8,9), but not FGFR1 (a3,4). **b**. Representative immunohistochemistry for Cytokeratin-5 (KRT5) (b1,2), Fascin (b3,4), FGF1 (b5,6) α-SMA (b7,8), FGFR1 (b9,10), and FGFR2 (b11,12) in serial sections of IPF lung tissue. In IPF, immunostaining for FGF1, FGFR1 and FGFR2 was observed in myofibroblasts of fibroblast foci [FF] (indicated by arrowheads and α-SMA-staining) as well as in overlying hyperplastic bronchiolar basal cells (indicated by asterisks and KRT5-staining), and colocalized with expression of the migratory marker Fascin (2). Representative immunohistochemistry for α-SMA (b13), FGFR3 (b14) and FGFR4 (b15) and FGF1 (b16) in serial sections of IPF lung tissue. In general, α-SMA-positive myofibroblasts of FF (indicated by arrowheads) express FGF1, FGFR3 and FGFR4. Of note, FGFR3 expression appeared predominantly nuclear in AECII as well as myofibroblastic cells. Scale bars: a1-9 (100 μm); b1,3,5,7,9,11 (250 μm), b2,4,6,8,10,12 (100 μm), b13-16 (100 μm)

In IPF lungs, FGF1 was present in SpC+, hyperplastic AECII cells, overlying regions of fibrosis (Fig. 3; a1-2). No or only weak FGFR1 staining was observed (Fig. 3; a3-4). FGFR2 was strongly expressed by proSP-C+ alveolar epithelium (Fig. 3; a5). as well as FGFR3 and FGFR4 (Fig. 3; a7 and a9, respectively). Alveolar macrophages also stained strongly for FGF1 as well as for FGFR2 and 4. FGFR1 and 3 staining of macrophages was faint (Additional file 3: Figure S2).

One characteristic phenotype of IPF lung architecture is the abnormal proliferation of basal cells (Keratin-5+) which assemble into ‘sheets’, [25] (Fig. 3, b1,2). In addition, α-SMA+, spindle-shaped myofibroblasts comprise usual interstitial fibrotic foci (FF) particular to IPF (Fig. 3; b7,8). The cytoskeleton actin-bundling protein Fascin [22, 26] was also used to identify potentially migrating cells of the FF (Fig. 3, b3,4). Basal cells were positive for Fascin (Fig. 3; b1,2 vs. b3,4) as well as many α-SMA positive fibroblasts (Fig. 3; b3,4 vs. b7,8). FGF1 was expressed by Keratin-5, Fascin, and α-SMA positive cells (Fig. 3; b5,6 vs. b1,2; b3,4 and b7,8 respectively). A similar pattern was observed for both FGFR1 (Fig. 3; B9,10) and FGFR2 (Fig. 3; B11,12) though FGFR2 was more strongly expressed overall than FGFR1 (See also Additional file 4: Figure S3). α-SMA positive fibroblasts also express FGFR3 and FGFR4 (Fig. 3, b13-16)

**Fig. 4:**
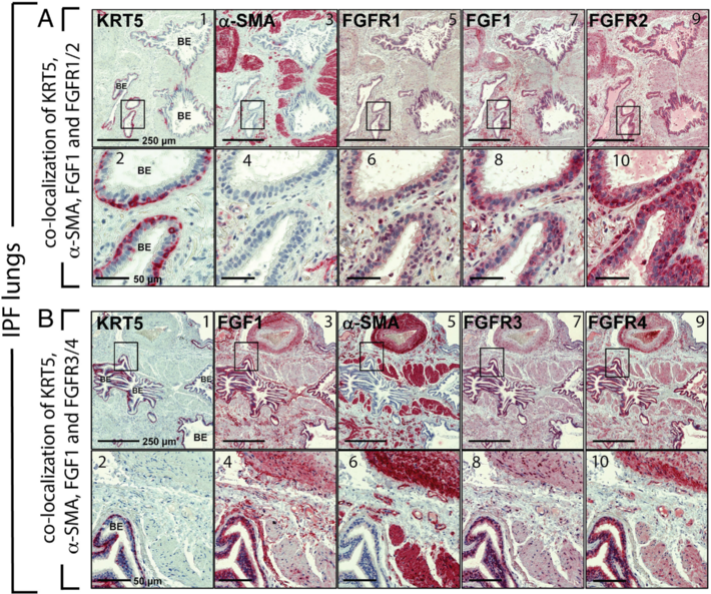
Overexpression of FGF1 and FGF-receptors FGFR1, FGFR2, FGFR3 and FGFR4 in hyperplastic bronchioles in remodelled areas of dense fibrosis in idiopathic pulmonary fibrosis (IPF) lungs. **a** Immunohistochemistry for KRT5 (a1,2), α-SMA (a3,4), FGFR1 (a5,6), FGF1 (a7,8) and FGFR2 (a9,10) in serial sections of IPF lung tissue. Moderate immunostaining for FGFR1, but very strong staining for FGF1and FGFR2 is observed in basal and luminal bronchial epithelial cells of abnormal, hyperplastic bronchioles (indicated by KRT5-staining) surrounded by dense fibrotic regions. **b** Representative immunohistochemistry for KRT5 (b1,2), FGF1 (b3,4), α-SMA (b5,6), FGFR3 (b7,8) and FGFR4 (b9,10) in serial sections of IPF lung tissue. In IPF, bronchial epithelial cells of abnormal bronchioles in areas of bronchiolization and dense fibrosis indicate robust expression of FGF1, FGFR3 and FGFR4. Scale bars: a1,3,5,7,9 (250 μm); a2,4,6,8,10 (50 μm), b1,3,5,7,9 (250 μm), b2,4,6,8,10 (50 μm)

**Fig. 5:**
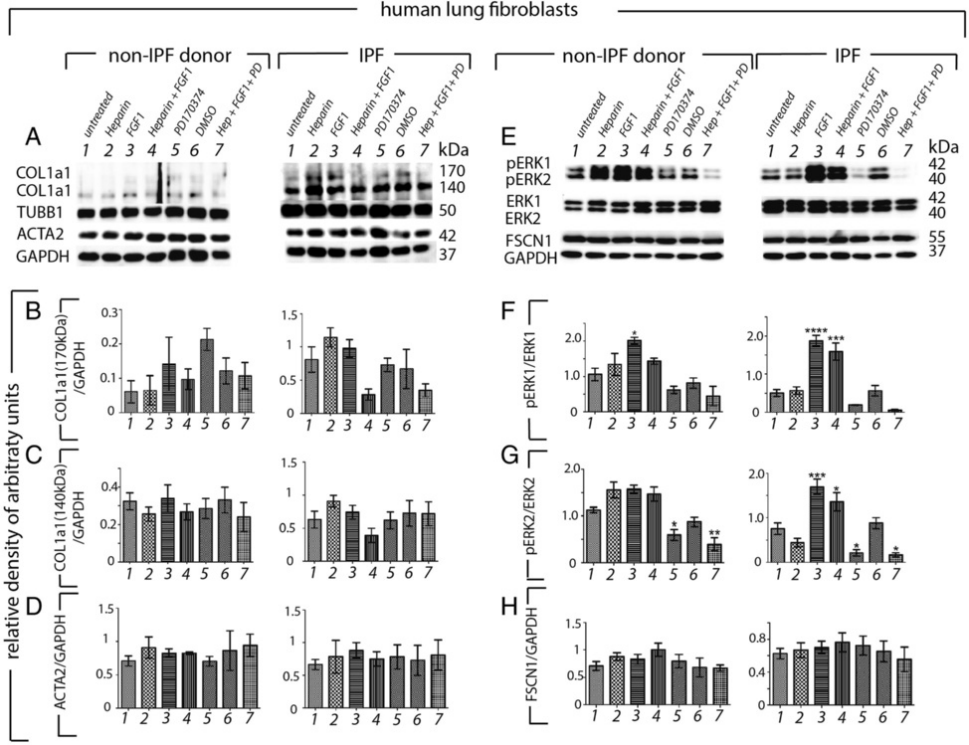
Impact of exogenous FGF1 + heparin on the regulation of pro-fibrotic proteins. Western blots were performed on lysates harvested from IPF and non-IPF (donor) fibroblasts (2 technical replicates of 6 independent biological samples). Cells were starved for 24 h (lane 1) and then treated once daily for two days with heparin (25 ng/mL) (lane 2) recombinant human FGF1 (25 ng/mL) (lane 3), FGF1 + heparin together (lane 4), the FGFR inhibitor, PD173074 (0.1 μM resuspended in DMSO) (lane 5), DMSO (0.1 μM) only (lane 6), or FGF1 + heparin + PD173074 (lane 7) simultaneously. Collagen 1a1 (COL1a1, bands present at both 170 and 140kDA) was blotted against b-tubulin (TUBB1) (**a**) and smooth muscle actin (ACTA2/α-SMA) was blotted against GAPDH (**b**). Densitometry plots of arbitrary units indicated that in donor fibroblasts, COL1a1 was not significantly regulated (**b**,**c**) and neither was ACTA2 (α-SMA) (**a**,**d**). Untreated lysates of IPF fibroblasts displayed more collagen than in donor controls (**a**). COL1a1, especially the 170kDA band, was strongly reduced in FGF1 + heparin groups (**b**,**c**) while ACTA2 was not regulated (**a**,**d**). Next p-ERK1/2 was blotted over total ERK (**e**,**f**,**g**), and Fascin (**e**,**h**), a cell invasion/migration marker. In donor fibroblasts, p-ERK1 signal was significantly increased in the FGF1 alone group and reduced in the presence of the inhibitor (**e**,**f**). The p-ERK2 signal was increased in donor fibroblasts where exogenous FGF1 or heparin or both were added and p-ERK2 was attenuated in the presence of the inhibitor (**e**,**g**). In IPF fibroblasts, p-ERK1 was significantly activated when exposed to FGF1 alone or FGF1 + heparin and attenuated when the inhibitor was present (**e**,**f**) and p-ERK2 was similarly regulated (**e**,**g**). A trend towards an increase in the cell migration marker Fascin was observed in FGF1 + heparin treated donor and IPF fibroblasts (**e**, **h**)

**Fig. 6:**
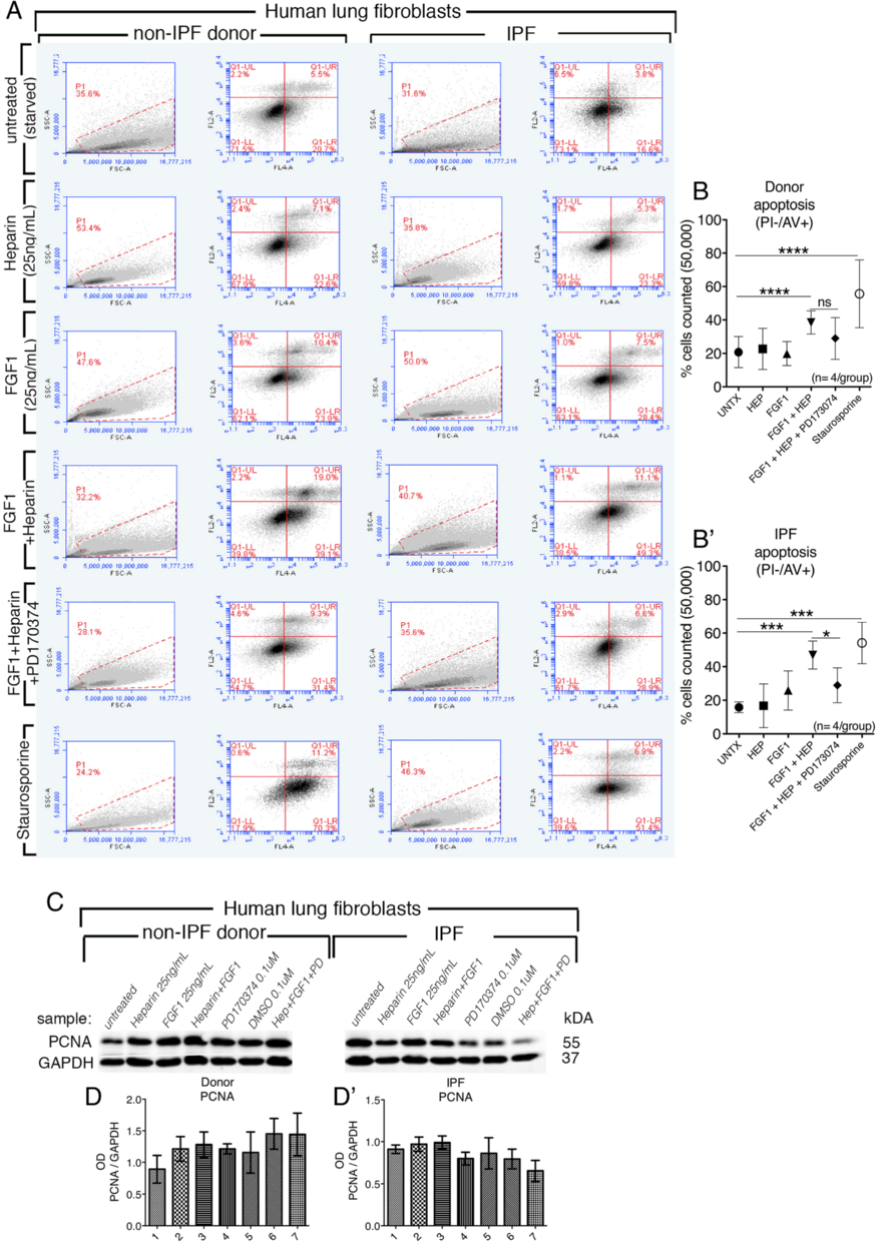
Impact of exogenous FGF1 on cell death and cell proliferation on fibroblasts from IPF and donor lungs. FACS plots (**a**) represent flow cytometry performed on treated donor and IPF fibroblasts for Annexin V (detected with the FL4A channel) and propidium iodide (detected with the FL2A channel). Cells were starved for 24 h and then treated once daily for two days with heparin (25 ng/mL) recombinant human FGF1 (25 ng/mL), FGF1 + heparin together, FGF1 + heparin + PD173074 (0.1 μM resuspended in DMSO), or as positive control 1uM Staurosporine (resuspended in DMSO) 18 h before harvest for experiment. Graphic representation of the percentage of apoptotic cells (Annexin V positive and propidium iodide negative) for donor fibroblasts (20 % vs. 40 %) (**b**) and IPF fibroblasts (18 % vs. 50 %) (**b’**); *n* = 4 biological samples/treatment group. The gating strategy is shown in Additional file 6: Figure S5. Western blot for proliferating cell nuclear antigen (PCNA) against GAPDH (**c**). Densitometry plots of arbitrary units indicated no significant regulation of PCNA in various treatment groups (**d**,**d**’)

**Fig. 7:**
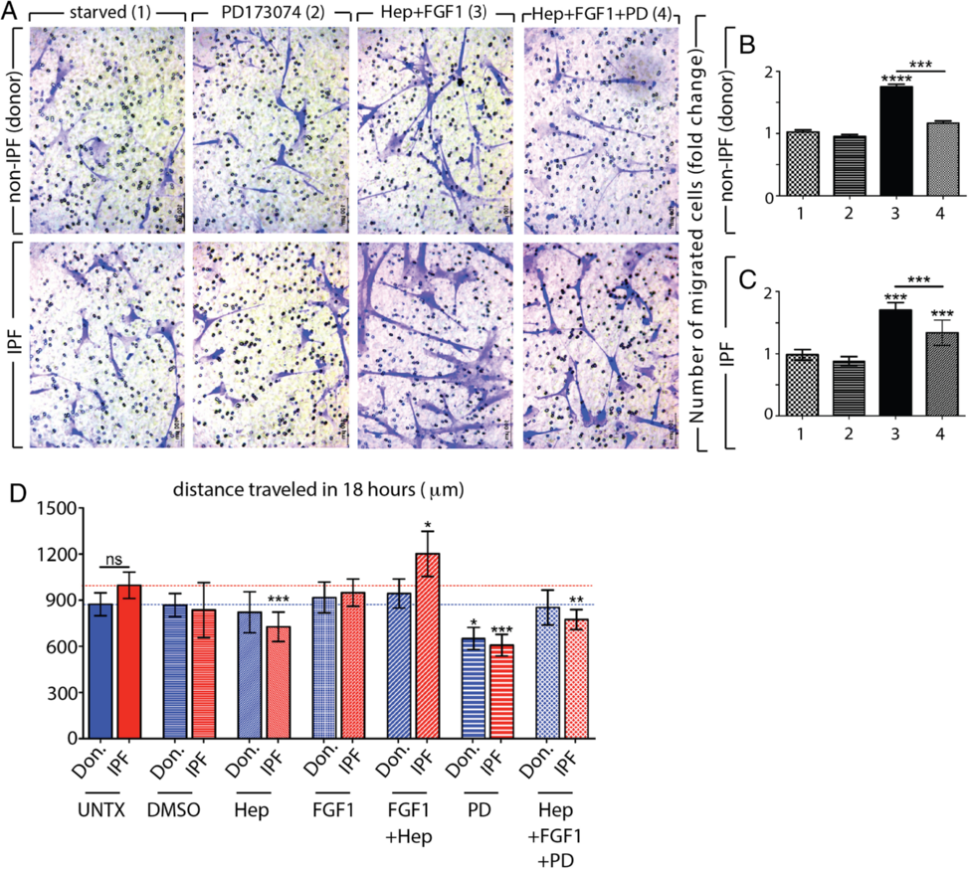
Impact of exogenous FGF1 on cell migration of IPF vs. Donor fibroblasts. Primary lung fibroblasts were starved for 24 h and seeded (12,000 cells/well) in the upper chamber of the transwell (6.5-mm transwell inserts with 8.0-μm pore size). Untreated (group 1) PD173074 (0.1 μM), (group 2), recombinant human FGF1 + heparin (25 ng/mL each) (group 3) or FGF1 + heparin + PD173074 (group 4) was added to the lower wells. Cells migrated for 16 h, were fixed and stained with crystal violet (**a**). The fold change of the number of migrated donor fibroblasts (**b**) and IPF fibroblasts (**c**). In both cases, PD173074 alone had no effect, FGF1 + heparin stimulated migration and and migration was attenuated in the FGF1 + heparin + PD173074 groups. Additional control experiments are available in Additional file 7: Figure S6. Graphs represent MetaMorph analyses of fibroblasts cultures for 18 h; blue = donor, red = IPF; total distance traveled in 18 h (**d**). FGF1 + heparin stimulated IPF fibroblasts to travel longer distances while no effect was observed in non-IPF, donor fibroblasts. Experiments were repeated in triplicate. Scale bars: (100 μm)

Keratin 5-staining was also used to identify bronchial epithelium of IPF lungs (Fig. 4a; 1,2 and Fig. 4b; 1,2). In addition, α-SMA+ was detected in highly condensed regions of smooth muscle cells (swhich surrounding the bronchioles) that are phenotypically distinct from the long, spindle-shaped α-SMA+ myofibroblastic cells of usual FF lesions which will be referred to in this manuscript as regions of “dense” smooth muscle (Fig. 4; a3 and b5,6). In the bronchial epithelium, FGFR1 lightly stained some bronchial epithelial cells (Fig. 4a; 5,6). In contrast to α-SMA+ myofibroblasts of usual FF regions, FGF1 was mostly absent in regions of ‘condensed’ smooth muscle (Fig. 4a; 7,8 and Fig. 4b; 3,4). FGFR2 was the most strongly expressed (Fig. 4a; 9,10), FGFR3 (Fig. 4b; 7,8) was nuclear, and FGFR4 was strongly present in the bronchial epithelium cytoplasm, and revealed also notable, but moderate expression in the dense smooth muscle (Fig. 4b; 9,10). Lastly, von Willebrand factor (vWF) positive endothelial cells did not show co-localization with FGF1, FGFR1 or FGFR2 staining (Additional file 5: Figure S4, A,B).

Given the strong expression of FGF1 and FGFR1/2/3/4 in regions of usual interstitial FF, as well as their co-localization with the migratory marker Fascin, and the myofibroblast marker α-SMA, the effect of exogenous FGF1 on IPF lung fibroblasts was addressed.

### FGF1 + heparin treatment of IPF and donor fibroblasts resulted in activation of the MAPK pathway and reduced COL1a1 production

IPF and non-IPF, donor fibroblasts (2 technical replicates of 6 independent biological samples) were harvested and cultured as previously described [27]. Cells were starved for 24 h and then treated once daily for two days with culture medium alone (line 1) heparin (25 ng/mL, line 2), recombinant human FGF1 (25 ng/mL, line 3), heparin + FGF1 together (line 4), the FGFR inhibitor, PD173074 (0.1 μM resuspended in DMSO, line 5), DMSO (0.1 μM) only (line 6), or heparin + FGF1 + PD173074 inhibitor simultaneously (line 7).

Heparin + FGF1 treatment of IPF fibroblasts resulted in a trend towards decreased collagen production by IPF fibroblasts while no effect was observed on non-IPF fibroblasts (Fig. 5a–c, compare line 4 to line 1). In the presence of the inhibitor, the reduction of collagen was partially attenuated. Contrary to previous reports using fibroblast cell lines, heparin + FGF1 did not significantly decrease α-SMA production (Fig. 5a,d).

The trend towards decreased collagen production in IPF fibroblasts treated with heparin + FGF1 may be in part regulated via activation of p-ERK1/2 signaling. The p-ERK1/2 signal was significantly increased in both FGF1 alone and FGF1 + heparin treated IPF fibroblasts (Fig. 5e,f,g). The p-ERK2 signal was significantly attenuated in fibroblasts treated with inhibitor alone versus untreated control, suggesting a high level of cell autonomous FGF-FGFR-signaling by IPF fibroblasts. In addition, the inhibitor efficiently blocked p-ERK activation by exogenous heparin + FGF1 in both donor and IPF fibroblasts (Fig. 5e,f,g). DMSO had no effect. A trend towards an increase in the cell migration marker Fascin was observed in heparin + FGF1 treated fibroblasts (Fig. 5e,h). However, this marker was not regulated following the addition of the FGFR inhibitor.

In summary, the FGFR inhibitor PD173074 efficiently mitigated both endogenous and exogenous FGFR mediated p-ERK signaling. Furthermore, FGF1 + heparin did not significantly attenuate α-SMA production nor did it significantly regulate Fascin. However, COL1a1 production trended towards a decrease in this group. Due to the substantial activation of p-ERK signaling following heparin + FGF1 treatment, whether proliferation and apoptosis were influenced was tested next.

### Heparin + FGF1 treatment induced apoptosis but did not affect proliferation

Flow cytometry was performed on treated donor and IPF fibroblasts for Annexin V and propidium iodide. In accordance with previous reports [20, 28], the number of apoptotic cells (Annexin V positive and propidium iodide negative) was increased in IPF and donor cells treated with heparin + FGF1 compared to starved, non-treated controls: 20 % vs. 40 % in donor fibroblasts (Fig. 6a,b) and 18 % vs. 50 % in IPF fibroblasts (Fig. 6a,b’). Apoptosis due to heparin + FGF1 treatment was partially mitigated in the presence of the FGFR inhibitor (Fig. 6a,b,b’). The gating strategy is shown in Additional file 6: Figure S5. As in previous studies performed on fibroblast cell lines [20], no significant change in the expression of proliferating cell nuclear antigen (PCNA) was detected in donor or IPF fibroblasts (Fig. 6c,d,d’).

### Heparin + FGF1 treatment stimulated migration of donor and IPF fibroblasts

To further investigate potential functional effects of exogenous FGF1 + heparin on primary lung donor and IPF fibroblasts, both transwell migration experiments and MetaMorph analyses of cell cultures were performed. The FGFR inhibitor alone (0.1 μM) did not inhibit migration through the transwell (Fig. 7a,b). The addition of heparin + FGF1 stimulated significant migration and when added in the presence of the FGFR inhibitor, this effect was mitigated (Fig. 7a,b,c). A control experiment showed that the FGFR inhibitor also attenuated migration stimulated by 5 % FCS (Additional file 7: Figure S6 A,B,C). While the addition of FGF1 alone or heparin alone did not stimulate migration in donor fibroblasts, these conditions stimulated migration in IPF fibroblasts, but to a lesser extent than heparin + FGF1 together (Additional file 7: Figure S6 D,E,F).

MetaMorph analyses of low-density fibroblasts cultures revealed that heparin + FGF1 stimulated IPF fibroblasts to travel longer distances than non-stimulated fibroblasts (Fig. 7d). In this system, addition of the inhibitor alone reduced distance travelled in both donor and IPF fibroblasts and mitigated the effect of exogenous heparin + FGF1 in IPF fibroblasts when added simultaneously. Unlike the transwell assay, FGF1 alone had no effect on IPF fibroblasts, while heparin alone reduced distance travelled.

In summary, heparin + FGF1 stimulated migration of both IPF and donor fibroblasts and this effect could be attenuated by the simultaneous addition of FGFR inhibitor.

## Discussion

### FGF1/FGFR expression is increased in IPF

In the rodent bleomycin-model, enhanced Fgfr2b-signaling on alveolar epithelial cells via the exogenous application or induction of FGF10 or FGF7, conferred increased survival and reduced lung fibrosis [7, 29]. On the other hand, attenuation of mesenchymal Fgfr2c-isoform signaling, led to decreased bleomycin-induced fibrosis [8]. These experiments suggest that in the context of lung fibrosis, a potential benefit is conferred via enhanced epithelial FGFR2b-signaling and decreased mesenchymal FGFR2c-signaling. This study is the first to describe the expression of FGFR2b-ligands (FGF1/7/10) and FGFR1/2/3/4 receptors in IPF. This study found that b-isoforms of FGFR1/2 receptors were decreased and c-isoform of FGFR1/2/3 were increased in IPF lungs suggesting an increase in FGF1/FGFR c-isoform signaling in IPF patients. However, the real contribution of endogenous FGFR-signaling to the pathogenesis of IPF is unknown. While it has been suggested that FGF-signaling contributes to increased angiogenesis in IPF via FGF2/FGFR2-signaling, the role of angiogenesis in IPF remains controversial and should be further studied [30, 31].

FGF1 emerged as the most highly expressed FGFR2b-binding ligand in IPF lung homogenates. FGF1 is expressed by both mesenchymal and epithelial cell types in the lung [9] and binds not only to FGFR2b, but with high affinity to all FGFRs [10]. The binding is also stabilized in the presence of heparin [32]. Whether FGF1 plays a pathogenic role in IPF, has not been thoroughly investigated. The hypoxic environment in IPF lungs may induce FGF1 expression, as FGF1 expression was shown to be strongly induced in rats exposed to hypoxia [33]. In addition, mast cells and basophils, whose numbers are increased in IPF, may provide a source of heparin [34] which in turn may augment FGF1 + heparin signaling on cells exposed to alveolar spaces. Furthermore, chronic obstructive pulmonary disease (COPD) is associated with enhanced bronchial expression of FGF1 and FGFR1 as well as FGF2 [35].

In this study, FGF1 and FGFR1/2/3 were increased at the protein level in whole lung homogenates taken from end-stage IPF patients compared to non-IPF lung homogenates. IHC analyses revealed robust expression of FGF1 in regions of irregular lung architecture particular to IPF, including: basal cell sheets or basal cells of hyperplastic bronchioles (Fig. 4a and b) and SMA+/Fascin + myofibroblasts of fibroblastic foci, and areas of thickened bronchial epithelium. Robust expression by macrophages was also observed. FGFR1 was faintly expressed in basal cells and myofibroblasts whereas FGFR2 was expressed very robustly in these cells, as well as also in SPC+ expressing alveolar epithelial type-II cells (AECII) and macrophages. FGFR3 was also highly expressed by AECII, bronchial cells, myofibroblasts and macrophages. Similarly, FGFR4 was expressed by nearly all epithelial cells and by myofibroblasts of fibroblastic foci. Taken together it is likely that FGF1-FGFR signaling is increased in regions of lung remodeling specific to IPF. Furthermore, given the reduction of p-ERK in fibroblasts treated with FGFR inhibitor, it is likely that FGF1/FGFR contributes to the overall increase in p-ERK1/2 and p-AKT signaling detected in whole lung homogenates which has also been previously reported [36]. Unchecked MAPK-signaling, in part sustained by increased FGF1/FGFR-signaling in IPF fibroblasts, may be a key mechanism by which activated fibroblasts persist in the fibrotic foci. Tyrosine kinase inhibitors such as Nintedanib, also known as BIBF1120 or Vargatef, block pathways that feed in to the MAPK pathway such as FGF, PDGFR and VEGF, which leads to reduced MAPK-signaling [37] and may be a mechanism by which this drug hinders disease progression.

### FGF1 + heparin treatment of IPF fibroblasts resulted in increased apoptosis and decreased collagen production but had no effect on smooth muscle actin

Former in vitro studies suggest that FGF1 may have an anti-fibrotic effect on lung fibroblasts. For example, heparin + FGF1 was found to decrease smooth muscle actin production and had a pro-apoptotic effect on a normal lung fibroblast cell line [20]. In addition, after induction of epithelial to mesenchymal transition (EMT) via Transforming growth factor β (TGF-β) stimulation of a human epithelial cell line (A549), FGF1 + heparin returned epithelial and mesenchymal markers to levels of non-stimulated cells [21]. Thus, heparin + FGF1 was capable of reversing TGF-β-mediated EMT via MAPK-dependent signaling. Similar results were obtained using the mouse lung epithelial cell line, MLE-12 (data not shown). In accordance with previous work, a decrease in COL1a1 production was observed by primary lung IPF fibroblasts exposed to heparin + FGF1. This result was not elicited in donor lung fibroblasts, as the level of collagen detected in donor fibroblasts was already very low. However, in contrast to previous in vitro studies performed on fibroblast cell lines, alpha smooth muscle actin (α-SMA) was not significantly regulated. The reduction in COL1a1 may be linked to the increase in p-ERK signaling which was elicited more strongly in IPF lung fibroblasts than donor lung fibroblasts. This stronger response by IPF lung fibroblasts may be due to distinct heparin sulfate proteoglycan formation on the IPF fibroblast cell surface, which may result in IPF fibroblasts being more amenable to heparin + FGF1 stimulation. Likewise, p-ERK signaling was inhibited by the addition of an FGFR inhibitor both alone and in the presence of exogenous heparin + FGF1. This strong increase in p-ERK signaling may also contribute to increased apoptosis of fibroblasts via MAPK-mediated activation of p38 [38, 39]. As previously observed in fibroblast cell lines [20, 40], both donor and IPF fibroblasts, heparin + FGF1 treatment resulted in increased apoptosis but no change in proliferation was observed.

### FGF1 + heparin treatment of IPF and non-IPF lung fibroblasts increased cell migration

FGF1 and FGFR co-localization with the motility and invasion marker Fascin was observed in usual interstitial fibroblastic foci. In addition, a preliminary scratch assay experiment revealed that FGF1 + heparin treated IPF fibroblasts closed a scratched area of confluent fibroblasts faster than untreated cells (data not shown). Although Fascin expression was not observed to be significantly regulated by heparin + FGF1, the failure of heparin + FGF1 to reduce SMA expression, the preliminary scratch assay data, the absence of FGF1 from condensed regions of smooth muscle cells, as well as previous studies indicating that FGF1 influences cell migration [41], was rational for further investigation.

The addition of heparin + FGF1 stimulated significant migration of both IPF and non-IPF fibroblasts through a transwell filter compared to starved, untreated cells. When heparin + FGF1 were added in the presence of the FGFR inhibitor, this effect was mitigated. Interestingly, while the addition of FGF1 alone or heparin alone did not stimulate migration in donor fibroblasts, these conditions stimulated migration in IPF fibroblasts, but to a lesser extent than heparin + FGF1 together. These results suggest that IPF fibroblasts may be primed to receive chemotactic signals. Faster migration by IPF fibroblasts may also be due to enhanced p-ERK1/2 signaling as stronger activation of p-ERK1/2 by FGF1 alone and heparin + FGF1 was also observed in IPF fibroblasts compared to donor, non-IPF fibroblasts used in this study.

In addition, MetaMorph analyses of low-density cultures of fibroblasts revealed that heparin + FGF1 stimulated IPF fibroblasts to travel longer distances while no effect was observed in non-IPF, donor fibroblasts. Unlike the transwell experiments, addition of the inhibitor alone reduced overall distance travelled in both donor and IPF fibroblasts compared to untreated cells. However, as in the transwell experiments, the FGFR inhibitor efficiently attenuated the effect of exogenous heparin + FGF1 in IPF fibroblasts when added simultaneously. Lastly, unlike the transwell assay, FGF1 alone had no effect on IPF fibroblasts, while heparin alone reduced distance travelled of IPF fibroblasts. The discrepancies between the transwell and live imaging experiments may be due the lack of a gradient formation in the 24-well plates used for live imaging. In summary, MetaMorph analyses of live imaging experiments support the conclusion that the migration of IPF fibroblasts was enhanced following exposure to heparin + FGF1.

### Increased FGF1-FGFR signaling may contribute to lung remodeling in IPF

In summary, this study described strong expression of FGF1 and FGFR1/2/3/4 receptors in pathogenic areas of IPF lungs and identified FGF1-FGFRs as potential contributors to increased MAPK-activity in IPF. Though IPF lung fibroblasts responded to heparin + FGF1 treatment by attenuating COL1a1 expression and increased apoptosis, increased p-ERK1/2 signaling along with enhanced cell migration was also observed reflecting a potentially dual nature of FGF1/FGFR in the context of lung fibrosis. Though tyrosine kinase inhibitors have recently been approved for the treatment of IPF, given the multi-faceted nature of FGF1-FGFR signaling, further studies should be designed to identify targets of growth factor signaling that mediate specific cellular functions such as fibroblast apoptosis and migration.

## Additional files

Additional file 1:
**Supplementary Methods.**
Additional file 2: Figure S1. Co-localization of FGF1, FGFR1/2/3/4 with macrophages (CD68+) in donor lung serial sections. Representative immunohistochemistry on serial sections of non-IPF (donor) lung tissue for FGFs and macrophages (CD68+). FGF1 (**A1,6**), FGFR2 (**A3,8**), FGFR3, (**A4,9**) and FGFR4 (**A5,10**), were detected in macrophages, but FGFR1 (**A2**,**7**) was not. All scale bars: 50 μm. Additional file 3: Figure S2. Co-localization of FGF1, FGFR1/2/3/4 with macrophages (CD68+) in IPF lung serial sections. Representative immunohistochemistry on serial sections of IPF lung tissue for FGFs and macrophages (CD68+). FGF1 (**A1,2**), FGFR2 (**A4,5**), FGFR3, (**A6,7**) and FGFR4 (**A8,9**), were detected in macrophages, but FGFR1 (**A2**,**7**) was not. All scale bars: 100 μm. Additional file 4: Figure S3. Co-localization on serial sections of FGF1, FGFR1/2 in usual fibroblastic foci (α-SMA) IPF lung lesion and basal cell sheet (KRT5) on serial sections. Representative immunohistochemistry on additional serial sections of IPF lung tissue for FGFs basal cell sheets (KRT5) and spindle-shaped myofibroblasts (α-SMA) of fibroblastic foci (FF). Representative of basal cell sheets KRT5 (**A1,6,11**), FGF1 (**A2,7,12**), α-SMA (**A3**,**8**,**11**), FGFR1 (**A4,9,14**) and FGFR2 (**A5,10,15**). FGFR2 and FGF1 were strongly expressed in fibroblasts of FFs and to a lesser extent FGFR1. Abnormal basal cell sheet covering fibroblastic foci (**B1**), (α-SMA) of FF (**B2**), FGF1 is present in myofibroblasts and in basal cell sheets (**B3**), and so is FGFR2 (**B4**). Scale bars: **A1-5** (500 μm); **A6-10** (250 μm), **A11-15; B1-4** (100 μm). Additional file 5: Figure S4. Co-localization on serial sections of FGF1, FGFR1/2 in areas of dense smooth muscle (α-SMA+) and vessels stained with von Willibrand Factor (vWF) in IPF lungs. Representative immunohistochemistry on serial sections of IPF lung tissue for FGFs relative t dense smooth muscle (α-SMA) and vessels (vWF). Representative of hyperplastic basal cells (KRT5+) (**A1,7,13**), dense smooth muscle α-SMA (**A2,8,14**), FGFR1 is absent in endothelium and lightly stains dense smooth muscle (**A3,9,15**). An additional series of sections showing that FGF1, FGFR1 and FGFR2 are mostly absent from endothelium (**B1-5**). Scale bars: **A1-6** (500 μm); **A7-12** (100 μm), **A13-18; B1-5** (50 μm). Additional file 6: Figure S5. Gating strategy for Annexin V (AV)/Propidium Iodide (PI) FACS. Gating was performed based on single stained PI only stained cells (**A**) and Annexin V only stained cells (**B**). Additional file 7: Figure S6. Impact of exogenous FGF1 on cell migration of IPF vs. donor fibroblasts. Primary lung fibroblasts were starved for 24 h and seeded (12,000 cells/well) in the upper chamber of the transwell (6.5-mm transwell inserts with 8.0-μm pore size). Cells migrated for 16 h, and were then fixed and stained with crystal violet. (n = 3/treatment group) (**C**,**F**). 5%FCS stimulated migration of donor fibroblasts (**A, C1-4**) and IPF fibroblasts (**B, C5-8**) and these effects were attenuated by the simultaneous addition of 0.1uM and 1.0uM of PD173074. The addition of heparin or FGF1 alone did not stimulate migration in donor fibroblasts, however the addition of both factors significantly stimulated migration (**D, F1-4**). In contrast, both heparin and FGF1 alone stimulated migration of IPF fibroblasts, but not as significantly as FGF1 + heparin together (**E, F5-8**). Scale bars: (100 μm).
