# Supplementary material for: Increased FGF1-FGFRc expression in idiopathic pulmonary fibrosis
Source: Respir Res. 2015 Jul 3;16(1):83. doi: 10.1186/s12931-015-0242-2 (PMC4495640; doi:10.1186/s12931-015-0242-2)
Supplement: Additional file 1: — Supplementary Methods. [file 12931_2015_242_MOESM1_ESM.docx]

**Supplementary Data**

**Increased FGF1-FGFRc expression in Idiopathic Pulmonary Fibrosis**

BreAnne MacKenzie^1^, Martina Korfei^1^, Ingrid Henneke^1^, Zaneta Sibinska^1^, Xia Tian^1^, Stefanie Hezel^1^, Salma Dilai, Roxana Wasnick^1^, Beate Schneider^1^, Jochen Wilhelm^1^, Elie El Agha^1^, Walter Klepetko^2^, Werner Seeger^1,3^, Ralph Schermuly^1,3^, Andreas Günther^1,3,4^ and Saverio Bellusci^1,3,5,6 #^

^1^German Center for Lung Research, Excellence Cluster Cardio-Pulmonary System, Universities of Giessen and Marburg Lung Center, Giessen, Hessen, Germany.

^2^Dept of Thoracic Surgery, General Hospital University Vienna, Austria

^3^Member of the German Center for Lung Research

^4^AGAPLESION Lung Clinic Waldhof-Elgershausen, Greifenstein, Germany

^5^Developmental Biology Program, Division of Surgery, Saban Research Institute of Children's Hospital Los Angeles, University of Southern California Keck School of Medicine, Los Angeles, California

^6^Institute of Fundamental Medicine and Biology, Kazan (Volga Region) Federal University, 18 Kremlyovskaya Street, Kazan 420008, Russian Federation

^#^ Corresponding author

Saverio.Bellusci@innere.med.uni-giessen.de

**Supplementary Methods**

**Table 1: Antibodies used for western blots**

| **Primary Antibody (human)** | **Company/ Catalog** # | **Dilution** | **Secondary Antibody (human)** | **Company/ Catalog**# |
| --- | --- | --- | --- | --- |
| ACTA2 | **Abcam**  **(ab119952)** | **1:5000** | **Rabbit anti mouse HRP** | **Dako (**#P0260) |
| COL1a1 | **Rockland (**#600-401-103) | **1:1000** | Swine anti rabbit HRP | **Dako (**#P0217) |
| Fascin | **Abcam**  **(ab49815)** | **1:1000** | **Rabbit anti mouse HRP** | **Dako (**#P0260) |
| FGF1 | Abcam (ab-9588) | 1:200 | Rabbit anti goat HRP | Dako (#P0160) |
| FGF7 | Santa Cruz (# sc-27126) | 1:200 | Rabbit anti goat HRP | Dako (#P0160) |
| FGF10 | Santa Cruz (# sc-7375) | 1:200 | Swine anti rabbit HRP | Dako (#P0217) |
| FGFR1 | Santa Cruz (# sc-8318) | 1:200 | Swine anti rabbit HRP | Dako (#P0217) |
| FGFR2 | Abcam  (#10648) | 1:2000 | Swine anti rabbit HRP | Dako (#P0217) |
| FGFR3 | Abcam  (ab176459) | 1:500 | Swine anti rabbit HRP | Dako (#P0217) |
| FGFR4 | Abcam  (#ab 5481) | 1:500 | Swine anti rabbit HRP | Dako (#P0217) |
| p-ERK1/2 | cell signaling (# 4370S) | 1:1000 | Swine anti rabbit HRP | Dako (#P0217) |
| total-ERK1/2 | cell signaling (# 9102S) | 1:1000 | Swine anti rabbit HRP | Dako (#P0217) |
| p-AKT | cell signaling (# 4060S) | 1:1000 | Swine anti rabbit HRP | Dako (#P0217) |
| total-AKT | cell signaling (# 4691S) | 1:3000 | Swine anti rabbit HRP | Dako (#P0217) |
| β-Actin | Abcam (#ab8226) | 1:30000 | Rabbit anti mouse HRP | Dako (#P0260) |
| PCNA | Abcam  (#ab18197) | 1:500 | Swine anti rabbit HRP | Dako (#P0217) |
| β-Tubulin | Sigma (#T0198) | 1:1000 | Rabbit anti mouse HRP | Dako (#P0260) |

**Table 2: Human primers**

Primers were designed using Roche Universal Probe Library Assay design free online software.

| **Human Primers** | **Forward** | **Reverse** |
| --- | --- | --- |
| *ACTA2* | ctgttccagccatccttcat | tcatgatgctgttgtaggtggt |
| *COL1A1* | atgttcagctttgtggacctc | ctgtacgcaggtgattggtg |
| *FGF1* | caatgtttgggctaagacctg | ggctgtgaaggtggtgattt |
| *FGF10* | gaaggagaactgcccgtaca | ggcaacaactccgatttctact |
| *FGF7* | aagggacccaagagatgaaga | cctttgattgccacaattcc |
| *FGFR1b* | gcattcggggattaatagctc | ccacaggtctggtgacagtg |
| *FGFR2b* | gataaatagttccaatgcagaagtgct | tgccctatataattggagaccttaca |
| *FGFR3b* | CAAGTTTGGCAGCATCCGGCAGAC | TCTCAGCCACGCCTATGAAATTGGTG |
| *FGFR1c* | accaccgacaaagagatgga | gcagagtgatgggagagtcc |
| *FGFR2c* | TTCTCTTCCAGGCGCTGG | CTCTGCGTGGCTGGTGGTGC |
| *FGFR3* | CAAGTTTGGCAGCATCCGGCAGAC | CACCACCAGCCACGCAGAGTGATG |
| *FGFR4* | GCCCTTCACGTTATTGCAGATG | CCTCTCCAACCCCGTACTC |
| *PGBD* | tgtctggtaacggcaatgcg | cccacgcgaatcactctcat |

**Harvesting RNA from cells**

Human fibroblasts were grown in monolayers in 6 well plates to 80 – 100% confluency. Media was aspirated, cells were washed with 1X PBS briefly, and 200 ul of TRIZOL was added per well. Cells were scraped away with a cell scraper and pipetted into a cryovial. At least 3 wells were combined into one cryovial and vortexed for at least 30 s and placed immediately in liquid nitrogen. Homogenized cell lysates were then stored in -80°C until RNA was extracted. Heavy phase-lock gel tubes (5 PRIME 2302830) eppendorf tubes were centrifuged for 5 min at maximum speed. The homogenized lysate was added to the separator column and incubated at RT for 3 min. 1/10 total volume of chloroform was added. Tubes were shaken for at least 15 s and incubated for 3 min at RT. Next, tubes were centrifuged for 15 min at 10,000 g at 4°C. The aqueous layer was extracted and added to a new eppendorf. 1.5 volumes 100% isopropanol was added. Tubes were inverted to mix and incubated for 10 min at RT. After incubation, tubes were spun down for 15 min at 10,000 g, 4°C. After the pellet was identified, the supernatant was discarded and the pellet was washed with 75% EtOH and vortexed. Lastly, tubes were centrifuged for 5 min at 8,000 g, 4°C; supernatant was discarded, and pellet was dried on the bench until it became transparent. 30 ul of ddH2O was added and samples were Nanodropped for RNA concentration and purity.

**Table 3: Antibodies used for immunohistochemistry**

For immunostaining, the streptavidin-biotin-horse radish peroxidase (HRP) method with use of the ZytoChem-Plus AP Kit (Fast Red) (AP008RED-MS) was employed (Zytomed Systems, Berlin, Germany), according to a previously published protocol (S1).

| **Primary Antibody (human)** | **Company/ Catalog** # | **Dilution** |
| --- | --- | --- |
| cytokeratin-5 | Abcam  (ab75869) | 1:200 |
| Fascin | **Abcam**  **(ab49815)** | 1:100 |
| pro-SP-C | Millipore  (AB3786) | 1:750 |
| Von Willebrand Factor | Dako  (A0082) | 1:400 |
| Smooth Muscle Actin | Abcam  (ab5694) | 1:100 |
| FGF1 | Abcam (ab-9588) | 1:100 |
| FGFR1 | Santa Cruz (#sc-8318) | 1:50 |
| FGFR2 | Abcam  (#10648) | 1:100 |
| FGFR3 | Abcam  (ab176459) | 1:100 |
| FGFR4 | Abcam  (#ab5481) | 1:50 |

**Supplementary References:**

(S1) Korfei M, Schmitt S, Ruppert C, Henneke I, Markart P, Loeh B, Mahavadi P, Wygrecka M, Klepetko W, Fink L, Bonniaud P, Preissner KT, Lochnit G, Schaefer L, Seeger W, Guenther A. Comparative proteomic analysis of lung tissue from patients with idiopathic pulmonary fibrosis (IPF) and lung transplant donor lungs. *J Proteome Res* 2011;10:2185–205.
