# Supplementary figures and images for: Increased FGF1-FGFRc expression in idiopathic pulmonary fibrosis
Source: Respir Res. 2015 Jul 3;16(1):83. doi: 10.1186/s12931-015-0242-2 (PMC4495640; doi:10.1186/s12931-015-0242-2)

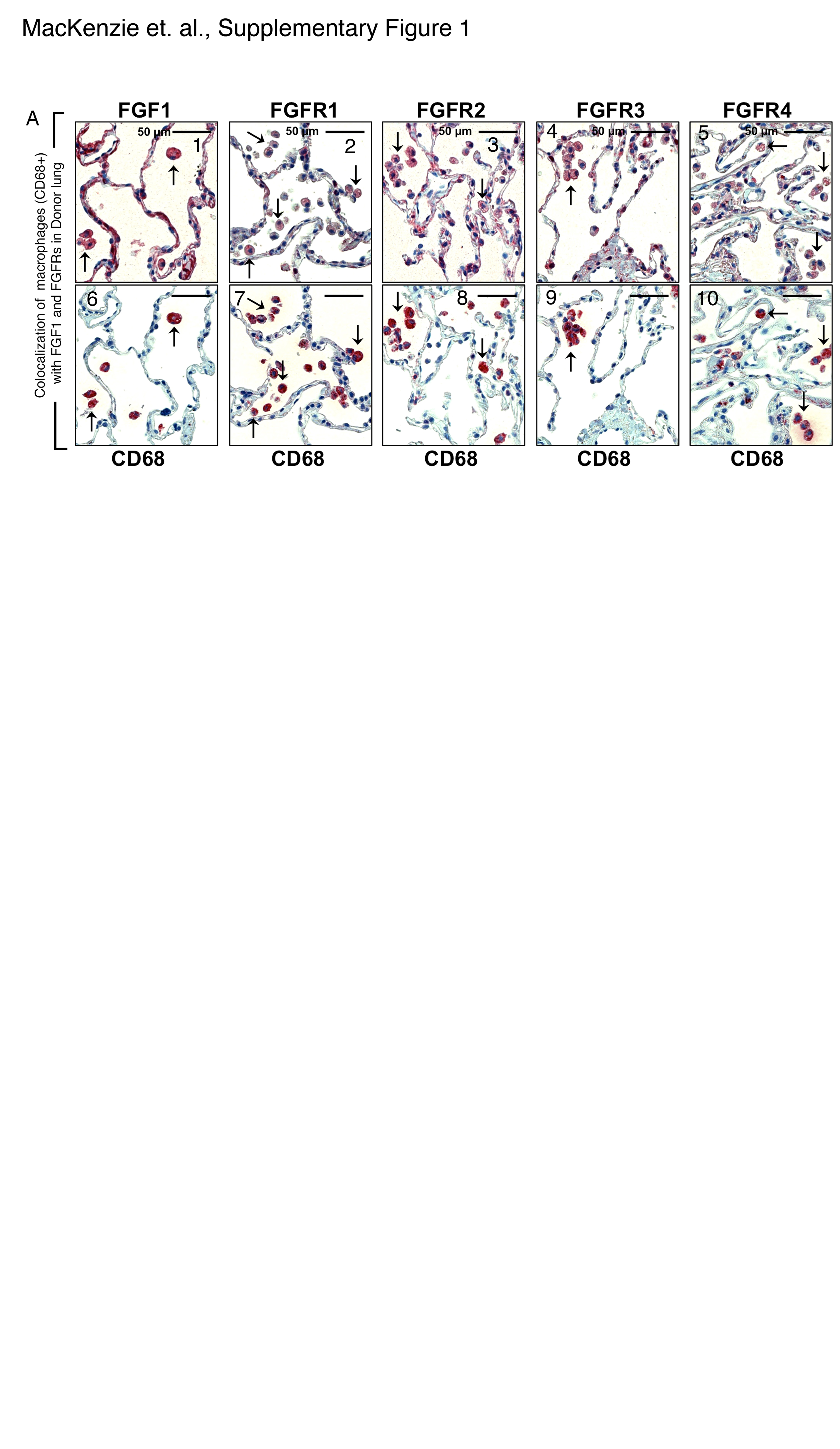

Supplement: Additional file 2: Figure S1. — Co-localization of FGF1, FGFR1/2/3/4 with macrophages (CD68+) in donor lung serial sections. Representative immunohistochemistry on serial sections of non-IPF (donor) lung tissue for FGFs and macrophages (CD68+). FGF1 (A1,6), FGFR2 (A3,8), FGFR3, (A4,9) and FGFR4 (A5,10), were detected in macrophages, but FGFR1 (A2,7) was not. All scale bars: 50 μm. [file 12931_2015_242_MOESM2_ESM.jpeg]

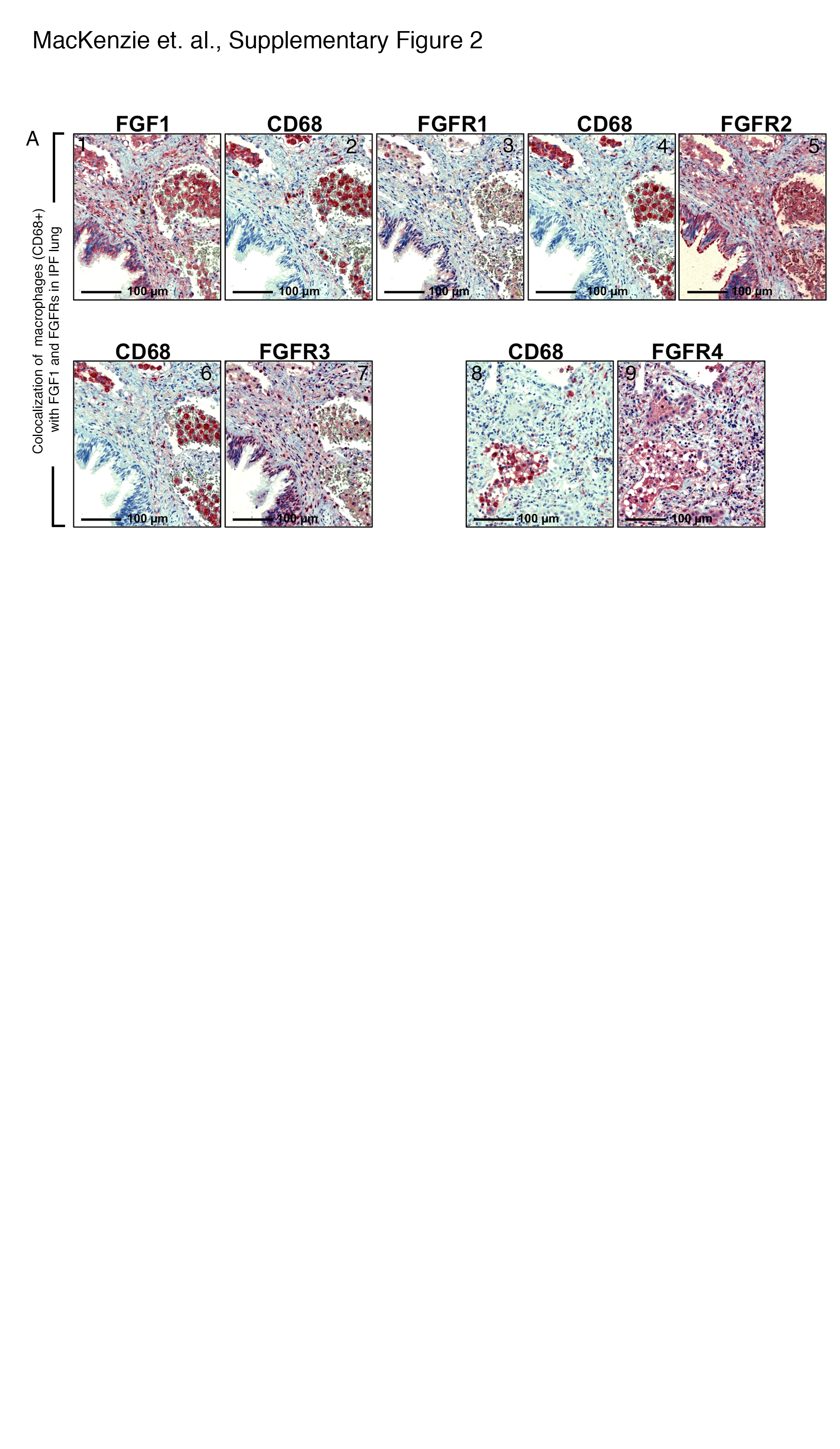

Supplement: Additional file 3: Figure S2. — Co-localization of FGF1, FGFR1/2/3/4 with macrophages (CD68+) in IPF lung serial sections. Representative immunohistochemistry on serial sections of IPF lung tissue for FGFs and macrophages (CD68+). FGF1 (A1,2), FGFR2 (A4,5), FGFR3, (A6,7) and FGFR4 (A8,9), were detected in macrophages, but FGFR1 (A2,7) was not. All scale bars: 100 μm. [file 12931_2015_242_MOESM3_ESM.jpeg]

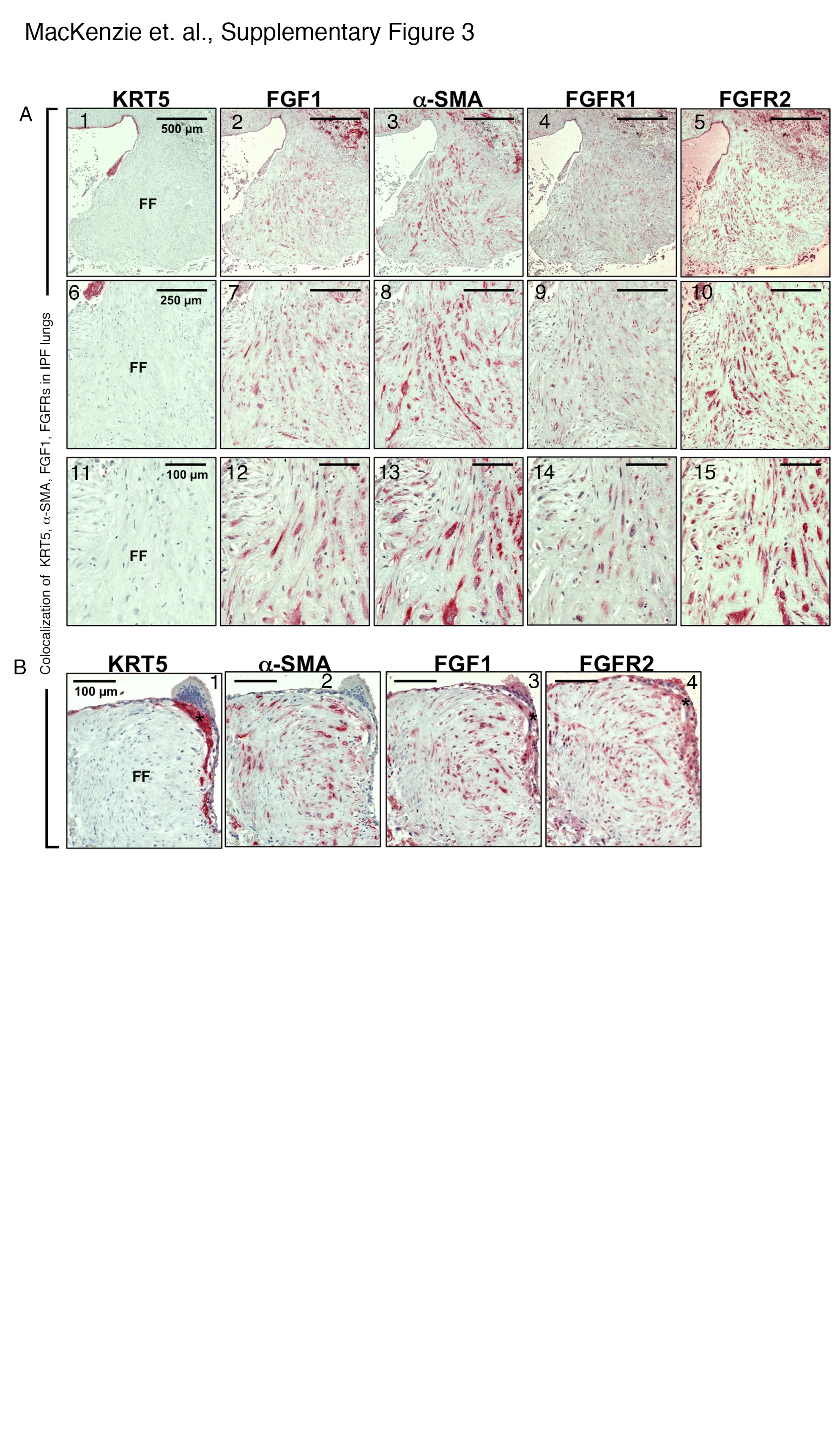

Supplement: Additional file 4: Figure S3. — Co-localization on serial sections of FGF1, FGFR1/2 in usual fibroblastic foci (α-SMA) IPF lung lesion and basal cell sheet (KRT5) on serial sections. Representative immunohistochemistry on additional serial sections of IPF lung tissue for FGFs basal cell sheets (KRT5) and spindle-shaped myofibroblasts (α-SMA) of fibroblastic foci (FF). Representative of basal cell sheets KRT5 (A1,6,11), FGF1 (A2,7,12), α-SMA (A3,8,11), FGFR1 (A4,9,14) and FGFR2 (A5,10,15). FGFR2 and FGF1 were strongly expressed in fibroblasts of FFs and to a lesser extent FGFR1. Abnormal basal cell sheet covering fibroblastic foci (B1), (α-SMA) of FF (B2), FGF1 is present in myofibroblasts and in basal cell sheets (B3), and so is FGFR2 (B4). Scale bars: A1-5 (500 μm); A6-10 (250 μm), A11-15; B1-4 (100 μm). [file 12931_2015_242_MOESM4_ESM.jpeg]

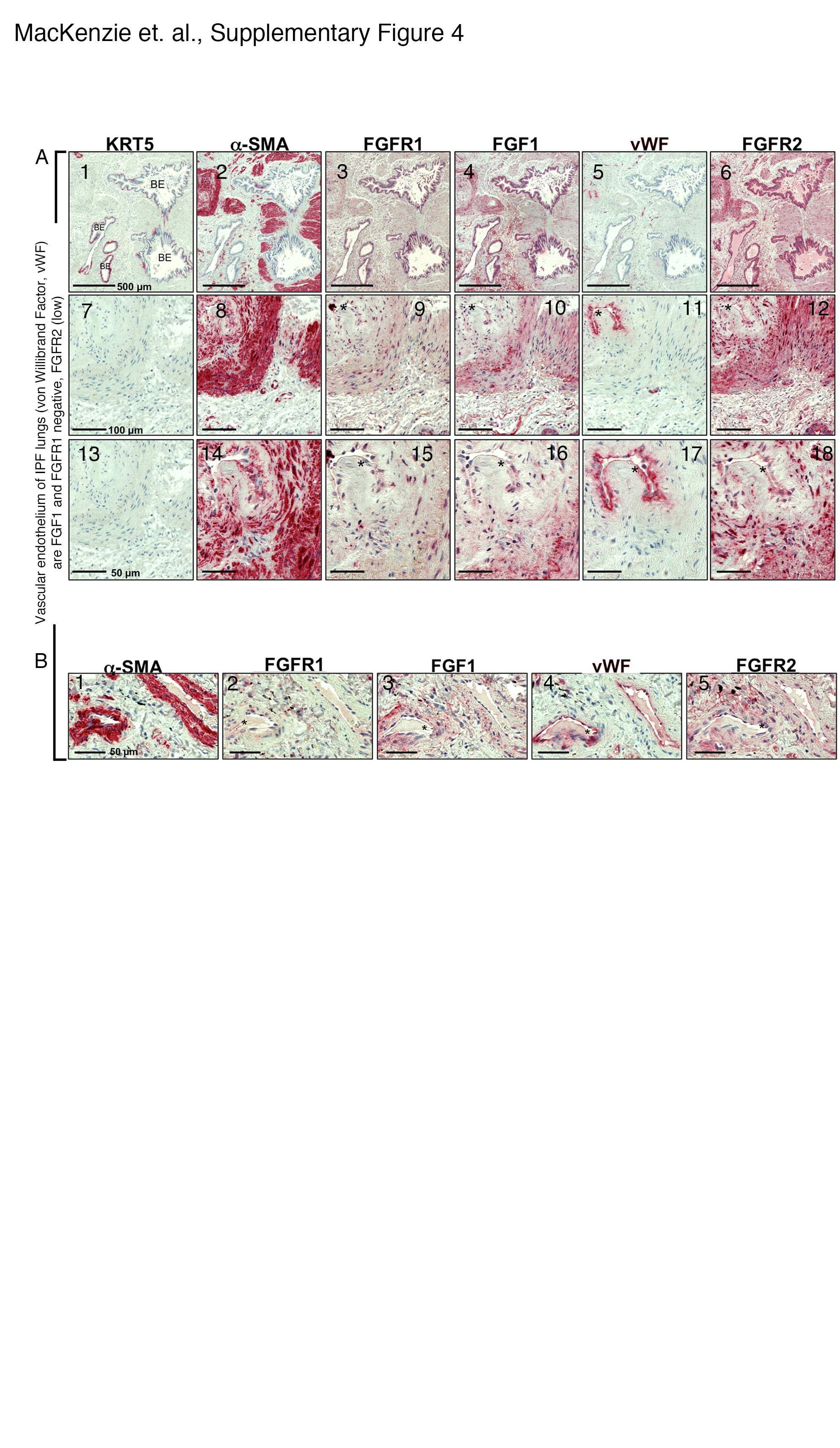

Supplement: Additional file 5: Figure S4. — Co-localization on serial sections of FGF1, FGFR1/2 in areas of dense smooth muscle (α-SMA+) and vessels stained with von Willibrand Factor (vWF) in IPF lungs. Representative immunohistochemistry on serial sections of IPF lung tissue for FGFs relative t dense smooth muscle (α-SMA) and vessels (vWF). Representative of hyperplastic basal cells (KRT5+) (A1,7,13), dense smooth muscle α-SMA (A2,8,14), FGFR1 is absent in endothelium and lightly stains dense smooth muscle (A3,9,15). An additional series of sections showing that FGF1, FGFR1 and FGFR2 are mostly absent from endothelium (B1-5). Scale bars: A1-6 (500 μm); A7-12 (100 μm), A13-18; B1-5 (50 μm). [file 12931_2015_242_MOESM5_ESM.jpeg]

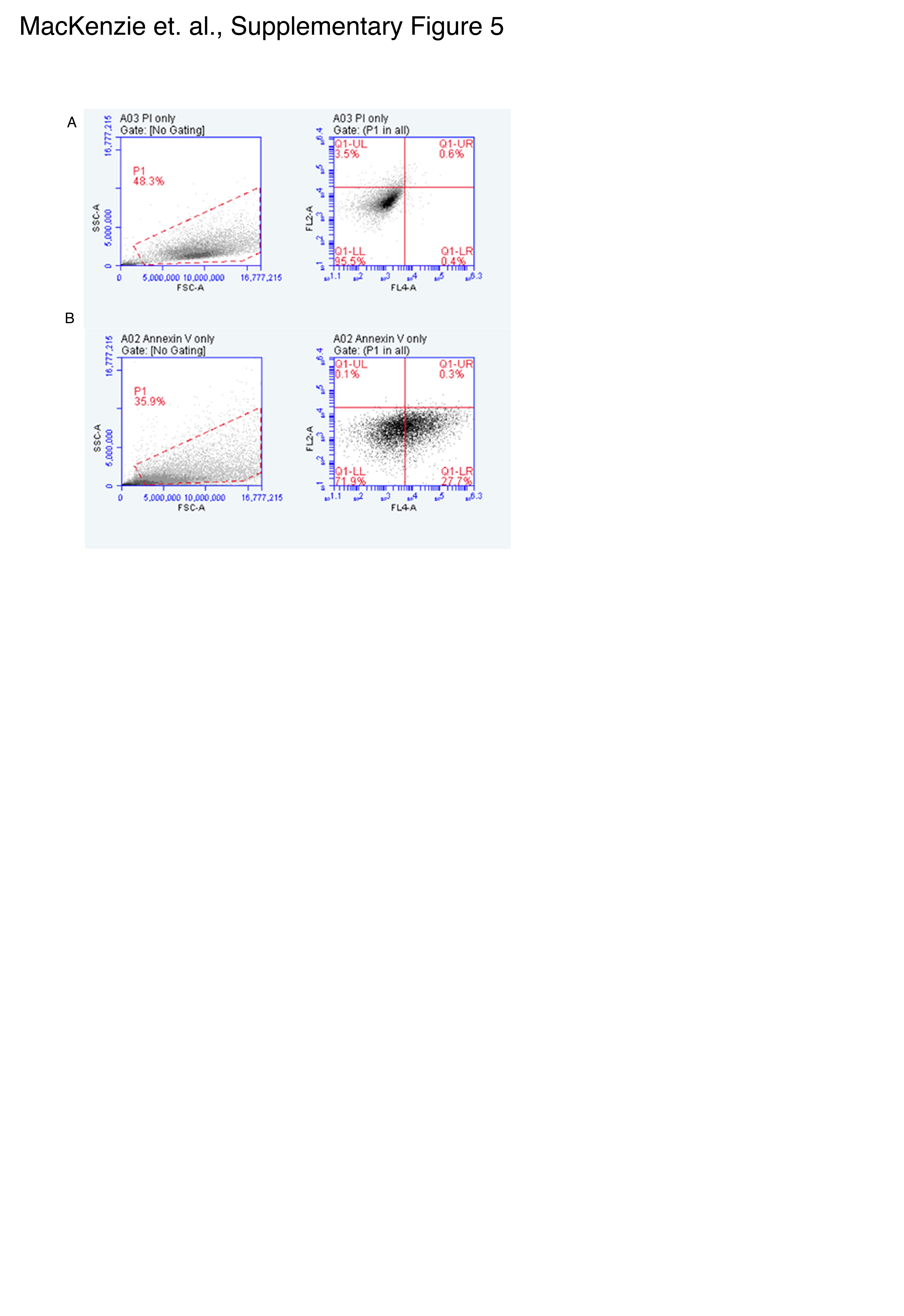

Supplement: Additional file 6: Figure S5. — Gating strategy for Annexin V (AV)/Propidium Iodide (PI) FACS. Gating was performed based on single stained PI only stained cells (A) and Annexin V only stained cells (B). [file 12931_2015_242_MOESM6_ESM.jpeg]

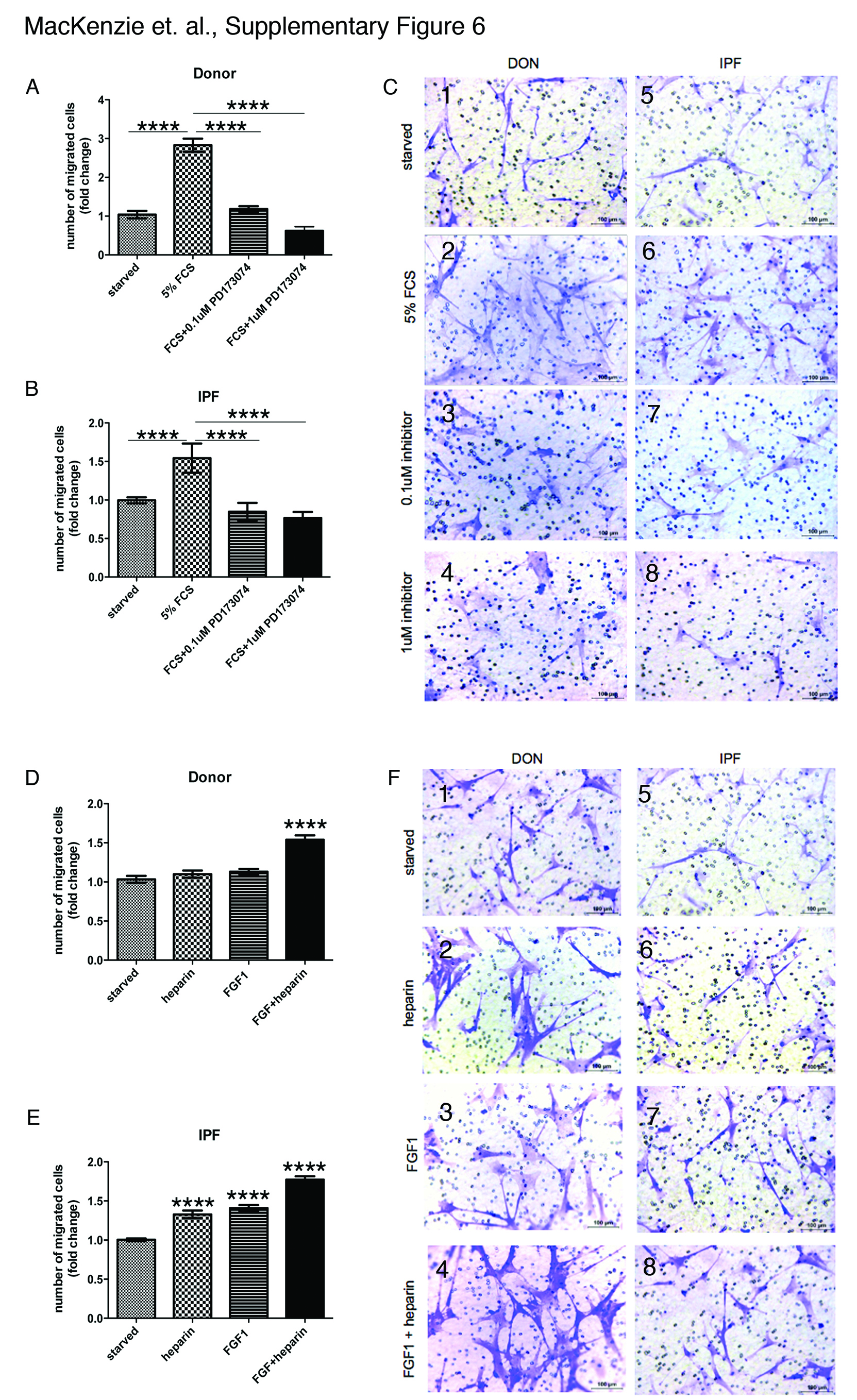

Supplement: Additional file 7: Figure S6. — Impact of exogenous FGF1 on cell migration of IPF vs. donor fibroblasts. Primary lung fibroblasts were starved for 24 h and seeded (12,000 cells/well) in the upper chamber of the transwell (6.5-mm transwell inserts with 8.0-μm pore size). Cells migrated for 16 h, and were then fixed and stained with crystal violet. (n = 3/treatment group) (C,F). 5%FCS stimulated migration of donor fibroblasts (A, C1-4) and IPF fibroblasts (B, C5-8) and these effects were attenuated by the simultaneous addition of 0.1uM and 1.0uM of PD173074. The addition of heparin or FGF1 alone did not stimulate migration in donor fibroblasts, however the addition of both factors significantly stimulated migration (D, F1-4). In contrast, both heparin and FGF1 alone stimulated migration of IPF fibroblasts, but not as significantly as FGF1 + heparin together (E, F5-8). Scale bars: (100 μm). [file 12931_2015_242_MOESM7_ESM.jpeg]
